# No Interaction with Alcohol Consumption, but Independent Effect of C12orf51 (HECTD4) on Type 2 Diabetes Mellitus in Korean Adults Aged 40-69 Years: The KoGES_Ansan and Ansung Study

**DOI:** 10.1371/journal.pone.0149321

**Published:** 2016-02-18

**Authors:** Jihye Kim, Bermseok Oh, Ji Eun Lim, Mi Kyung Kim

**Affiliations:** 1 Department of Preventive Medicine, College of Medicine, Hanyang University, Seoul, South Korea; 2 Institute for Health and Society, Hanyang University, Seoul, South Korea; 3 Department of Biochemistry and Molecular Biology, School of Medicine, Kyung Hee University, Seoul, South Korea; Hunter College, UNITED STATES

## Abstract

Previously, genetic polymorphisms of C12orf51 (HECTD4) (rs2074356 and/or rs11066280) have been shown to be related to alcohol consumption and type 2 diabetes (T2D). This study aimed to prospectively examine whether C12orf51 had an interaction with or independent effect on alcohol consumption and the risk of T2D. The present study included 3,244 men and 3,629 women aged 40 to 69 years who participated in the Korean Genome and Epidemiology Study (KoGES)_Ansan and Ansung Study. Cox proportional hazards models were used to estimate HRs and 95% CIs for T2D. rs2074356 and rs11066280 were associated with the risk of T2D after adjusting for alcohol consumption (rs2074356 for AA: HR = 0.39 and 95% CI = 0.17–0.87 in men, and HR = 0.36 and 95% CI = 0.13–0.96 in women; rs11066280 for AA: HR = 0.44 and 95% CI = 0.23–0.86 in men, and HR = 0.39 and 95% CI = 0.16–0.94 in women). We identified that the association of each variant (rs2074356 and rs11065756) in C12orf51 was nearly unchanged after adjusted for alcohol consumption. Therefore, the association of 2 SNPs in C12orf51 with diabetes may not be mediated by alcohol use. There was no interaction effect between alcohol consumption and the SNPs with T2D. However, even in never-drinkers, minor allele homozygote strongly influenced T2D risk reduction (rs2074356 for AA: HR = 0.35, 95% CI = 0.14–0.90, and *p*-trend = 0.0035 in men and HR = 0.34, 95% CI = 0.13–0.93, and *p*-trend = 0.2348 in women; rs11066280 for AA: HR = 0.36, 95% CI = 0.16–0.82, and *p*-trend = 0.0014 in men and HR = 0.39, 95% CI = 0.16–0.95, and *p*-trend = 0.3790 in women), while alcohol consumption did not influence the risk of T2D within each genotype. rs2074356 and rs11066280 in or near C12orf51, which is related to alcohol drinking behavior, may longitudinally decrease the risk of T2D, but not through regulation of alcohol consumption.

## Introduction

Diabetes is a major global health problem. It is estimated that 382 million people or 8.3% of adults worldwide had diabetes in 2013 [1]. In Korea, the prevalence of diabetes has increased from 1.5% in 1971 to 11.9% in 2013 [2, 3]. Although mortality related to diabetes has decreased in Korea [4], diabetes can cause several complications including nephropathy, neuropathy, and retinopathy, which are associated with increased medical costs [5].

The etiology of type 2 diabetes (T2D) is influenced by multi-factorial interplay between genetic and environmental factors including alcohol consumption. Moderate alcohol consumption is generally associated with reduced risk of diabetes [6]. This protective association is thought to be due to improved insulin sensitivity [7]. However, beneficial effects of alcohol consumption have not been consistent across studies. This discrepancy may be explained by different confounding structure between Asian and Western countries and, furthermore, could be explained by genetic mechanisms that regulate alcohol intake and metabolism [8].

Previously, studies on genetic polymorphisms in major alcohol-metabolizing enzyme genes such as alcohol dehydrogenases (ADH), aldehyde dehydrogenase (ALDH), and cytochrome P450 2E1 (CYP2E1) have been conducted [8]. Particularly, rs671 in ALDH2 is well known as a critical region related to alcohol drinking behavior [9–11]. Interestingly, two single nucleotide polymorphisms (SNPs) on chromosome 12q24.13 in or near C12orf51 (rs2074356 and/or rs11066280) associated with alcohol consumption have been reported in Koreans [12]. In the same population, there is a convincing cross-sectional association between rs2074356 in C12orf51 (*p* = 7.8x10^-12^) and biomedically important quantitative traits including waist-to-hip ratio [13]. The association of rs2074356 with the glycemic trait and T2D [14, 15] was also detected. The association between C12orf51 and alcohol consumption was interpreted by the possibility that signals in or near C12orf51 originate from a region of the ALDH2 gene, which encodes the hepatic enzyme, mitochondrial aldehyde dehydrogenase, and oxidizes acetaldehyde to acetate in alcohol metabolism [12]. The relationship between rs2074356 in or near C12orf51 and gamma-glutamyl transpeptidase (GGT) concentration may also be explained by the effect on alcohol consumption behavior [16]. rs2074356 and/or rs11066280 in or near C12orf51 were also associated with drinking behavior in Han Chinese [17].

These findings led us to question whether the association of two SNPs of C12orf51 with diabetes is modified by alcohol use, is mediated by alcohol use, or whether there is another pathway influencing T2D. In this study, we evaluated the prospective association between rs2074356 and rs11066280 in the C12orf51 gene and the risk of T2D and examined whether there was the interaction of these two SNPs with alcohol consumption or whether there was the association between SNPs and the risk of T2D unrelated to alcohol use by alcohol consumption-specific subgroup analysis among adults aged 40–69 years in a prospective cohort study, the Korean Genome and Epidemiology Study (KoGES)_Ansan and Ansung Study.

## Materials and Methods

### Study dataset

The study dataset was established with data from an ongoing community-based cohort that was part of the Korean Genome and Epidemiology Study (KoGES_Ansan and Ansung Study). Study participants were residents recruited from an industrialized community (Ansan city) located southwest of the capital city, Seoul and from a rural area (Ansung city) south of Seoul between 18 June 2001 and 29 January 2003. The cohort included a total of 10,038 individuals aged 40 to 69 years (5,018 participants from Ansung area and 5,020 participants from Ansan area). The details of the study design and procedures have been described previously [18].

After genotyping DNA samples and performing quality control, which have been described in detail elsewhere [13], a total of 8,842 participants were included in data analysis. Among 8,842 participants, those who did not complete responses regarding history taking in medicine (n = 146), had developed CAD, stroke, or cancer (n = 354), did not complete food frequency questionnaire (n = 248), or who reported implausible energy intake of < 500 kcal/day or > 4000 kcal/day) were excluded at baseline. Participants who reported taking diabetes medicine or who were identified as T2D by fasting plasma glucose (FPG) ≥ 7.0 mmol/l (126 mg/dl) or plasma glucose 2 h after ingestion of 75 g oral glucose load ≥ 11.1 mmol/l (200 mg/dl) at baseline were also excluded (n = 911). Participants who did not complete the questionnaire-based assessment of baseline alcohol consumption were additionally excluded (n = 151). Thus, the final group included 6,873 participants (3,244 men and 3,629 women). All procedures involving human subjects were approved by the Institutional Review Board of Kyung Hee University. Written informed consent was obtained from all subjects.

### General characteristics, anthropometrics, and biochemical variables

Participants completed questionnaires regarding demographics, medical history, and lifestyles, in addition to physical examinations and laboratory tests. Interview-based questionnaires were conducted to obtain demographic information (age, sex, residential area, marital status, and education), medical history (T2D, CAD, stroke, and cancer), family history of T2D, and lifestyle (smoking, alcohol drinking, and exercise). Anthropometric variables and blood pressure (systolic and diastolic blood pressure) were measured by trained researchers using standard methods. Weight was measured in kg to the nearest 0.01 kg and height was measured within 0.1 cm in light clothing without shoes. Body mass index (BMI) was calculated as weight (kg)/height (m^2^). Waist circumference (WC) was measured at a level midway between the lowest rib and the iliac crest. The average blood pressure value measured while lying down was used. After an 8- to 14-h fast, a blood sample was collected and the plasma concentrations of glucose, triglyceride, total cholesterol, and HDL-cholesterol were measured in a central laboratory.

### Alcohol consumption and dietary measurement

Participants were asked whether they had ever consumed at least 1 alcoholic drink every month, and if they had, they were asked whether they were former-drinkers or current -drinkers. In the case of current-drinkers, they were additionally asked about the amount of alcohol consumed in the last month. We calculated daily alcohol consumption using information on average frequency, amount per occasion, and the volume of 1 standard drink of alcohol. We classified participants into 4 groups: never-drinker, former-drinker, < 30 g/day, and ≥ 30 g/day using baseline alcohol consumption. However, alcohol consumption could have changed during follow-up, particularly after diagnosis of chronic diseases including T2D, cardiovascular diseases, or cancer and thus we calculated average alcohol consumption as being between baseline and before diagnosis of these diseases. In this analysis using average alcohol consumption, we classified participants into 4 groups: 0 g/day, > 0 and < 15 g/day, 15- < 30 g/day, and ≥ 30 g/day (S1 Table).

Dietary assessment was conducted using a semi-quantitative food frequency questionnaire (FFQ) with 103 food items at baseline, which was previously validated [19]. Nutrient intake was calculated by multiplying consumption frequency per day, portion size in grams of 103 food items, and nutrients per gram. Nutrients per gram were acquired from the seventh edition Food Composition Table of Korea published by The Korean Nutrition Society [20].

### Genotyping

Genomic DNA was genotyped on the Affymetrix Genome-Wide Human SNP Array 5.0. Bayesian robust linear modeling with the Mahalanobis distance (BRLMM) Genotyping Algorithm (Affymetrix) was used for the genotype calling of 500,568 SNPs. SNPs with missing genotype call rates >5%, minor allele frequency (MAF) <0.01, and Hardy-Weinberg equilibrium P<1×10^−6^ were eliminated before association analyses. The genotype calling and quality-control processes were described in more detail in a previous study [13]. rs2074356 and rs11066280 in or near *C12orf51* variants from the association analysis with alcohol consumption [12], glycemic traits [14, 15], or prevalent T2D [14] in the KoGES_Ansan and Ansung study were included in the following analyses. *C12orf51* (HECTD4) in chromosome 12 is known for its homologous to the E6-AP carboxyl terminus (HECT) domain containing E3 ubiquitin protein ligase 4. rs2074356 is located 47 kb downstream of C12orf51 and rs11066280 is located 2 kb upstream of C12orf51. In linkage disequilibrium (LD) analysis, rs2074356 and rs11066280 were in high LD (r^2^ = 0.83, D′ = 1.00).

### Follow-up and case ascertainment

Participants underwent a biennial follow-up including comprehensive health examination and questionnaire-based interviews to obtain updated information on their lifestyle factors and medical history and to ascertain case incidence of T2D. Using this information, a 10-year follow-up dataset between 2001 and 2010 was established. T2D cases were defined by the criteria of the World Health Organization (WHO) (FPG ≥ 7.0 mmol/l (126 mg/dl) or plasma glucose 2–h after ingestion of 75g oral glucose load ≥ 11.1mmol/l (200mg/dl)) [21] and self-reporting treatment by oral anti-diabetic drugs or insulin at re-examination or between examinations.

### Statistical analysis

Previous studies have reported the sex difference in the prevalence rates of diabetes [22, 23]. Men tend to have lower hepatic sensitivity to insulin and may, in consequence, have generally higher fasting levels of plasma glucose than women have [24]. In addition, because women have relatively low alcohol consumption, if sex-specific analysis was not conducted, characteristics of lower alcohol consumption group may depend on characteristic of women such as education, physical activity, diet, so on even after adjusting for sex. All analyses were conducted separately for men and women. Characteristics of men and women were compared using the Chi-square test for categorical variables and t-test for continuous variables. The average values and proportions of the baseline characteristics are presented. Potential confounders were identified by examining significant differences or linear trends across alcohol consumption groups using an age-adjusted general linear model. The linear trend test was performed by assigning the median value to each category of alcohol consumption and treating the value as a continuous variable. Iron intake was adjusted for total energy intake by using the residual method [25].

We calculated each participant’s person-time from baseline survey to the date of occurrence of T2D, loss to follow-up, or the end of the 5th survey in 2010, whichever came first. Hazard ratios (HRs) and 95% confidence intervals (CIs) were obtained by Cox proportional hazard models. The proportional hazards assumption in Cox regression was checked using an interaction term between the main exposure (alcohol consumption) and log of follow-up time (*P*>0.05). The relationships of two SNPs to FPG were identified in a cross-sectional study and therefore we confirmed whether they were related to incidence of T2D by the Cox proportional hazard model adjusted for age, residential area, and BMI. The interaction was evaluated by comparing nested models with and without interaction terms through the likelihood ratio test. In addition to linear trend across alcohol consumption groups, the other linear trend test for increasing frequency of minor allele was conducted in each alcohol consumption group using an additive model (1 degree of freedom). We conducted sensitivity analyses to assess the robustness of our results: we identified the interaction effects between alcohol consumption and SNPs 1) using average alcohol consumption to minimize measurement errors (S1 Table); 2) after censoring incident cases of CAD, stroke, or cancer during follow-up to test if our results were biased by changed diet or life style after developed severe diseases (S2 Table); and 3) after excluding the participants who did not follow up until 5th survey in 2010 to compare censored data with complete follow-up data (S3 Table). All statistical analyses were performed using the SAS software (version 9.3; SAS Institute, Cary, NC).

## Results

**Fig 1:**
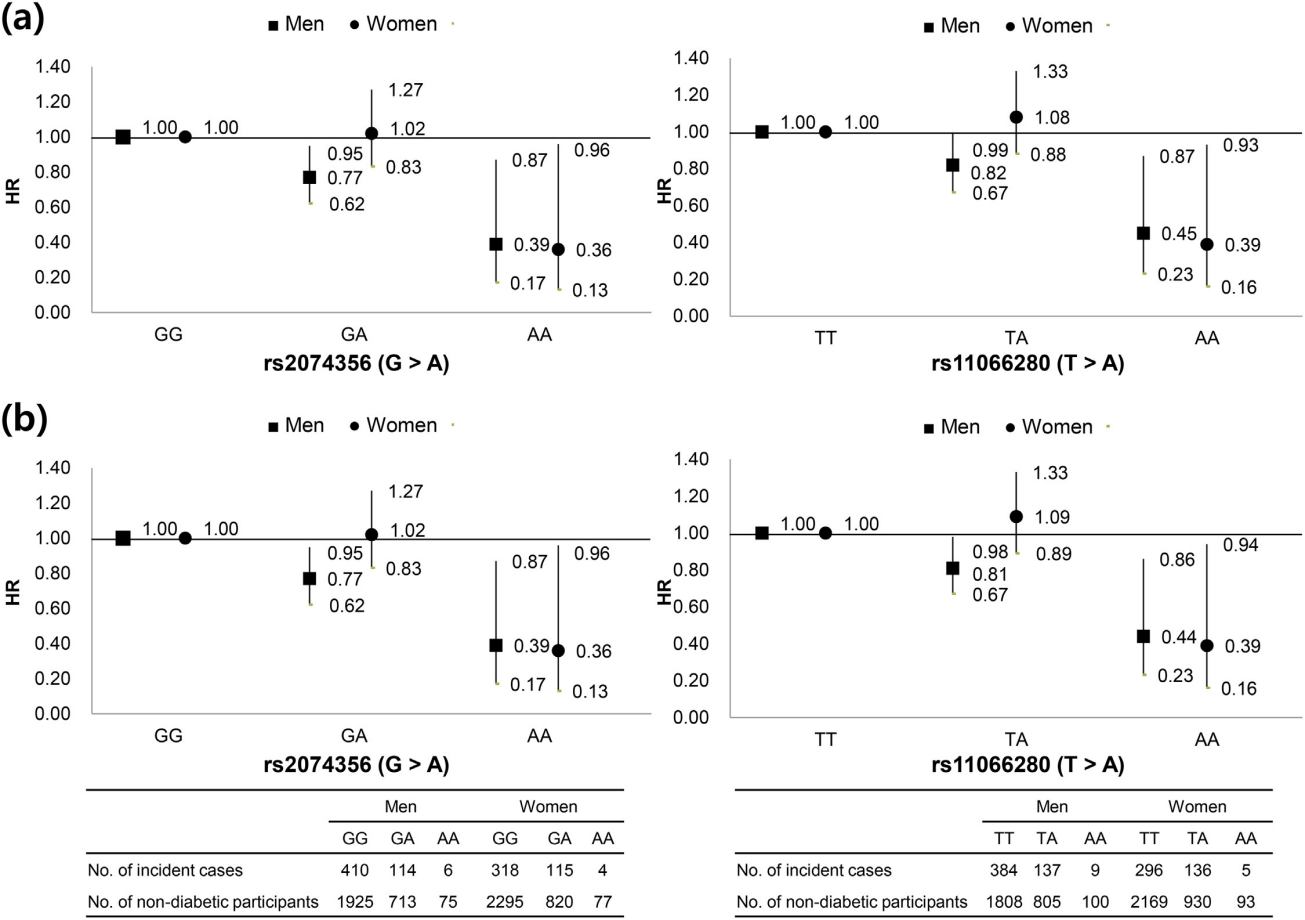
Hazard ratios and 95% CIs of type 2 diabetes according to SNP. Hazard ratios were calculated using a Cox proportional hazard model adjusted for age, residential area, and BMI at baseline.

A total of 6,873 participants (3,244 men and 3,629 women) were included in the present study. The general characteristics of the participants by sex are shown in Table 1. Men made up 47.2% of the total subjects and the mean ages of men and women were 50.8 and 51.8 years, respectively. A higher proportion of men were married, had a high school or above education, were drinkers and smokers, and had higher triglyceride, fasting blood glucose, and energy intake values in comparison to women. HRs and 95% CIs of T2D according to genotypes of two SNPs in C12orf51 are shown in Fig 1. rs2074356 and rs11066280 were associated with the risk of T2D after adjusting for alcohol consumption in both men and women (rs2074356 for AA: HR = 0.39 and 95% CI = 0.17–0.87 in men, and HR = 0.36 and 95% CI = 0.13–0.96 in women; rs11066280 for AA: HR = 0.44 and 95% CI = 0.23–0.86 in men, and HR = 0.39 and 95% CI = 0.16–0.94 in women). We identified that the association of each variant (rs2074356 and rs11065756) in C12orf51 was nearly unchanged after adjusted for alcohol consumption. Therefore, the association of 2 SNPs in C12orf51 with diabetes may not be mediated by alcohol use.

**Table 1:** General baseline characteristics of the study participants.

Characteristics	Men	Women	*p*^a^
n	3,244	3,629	
Age	50.8 ± 8.5^b^	51.8 ± 8.9	<0.0001
Residential area-Ansan (%)	61.3	51.9	<0.0001
Marital status-married (%)	96.2	86.1	<0.0001
Family history of diabetes (%)	9.5	11.3	0.0182
High school or beyond (%)	60.4	33.9	<0.0001
Former drinker (%)	9.5	3.0	<0.0001
Current drinker (%)	71.6	25.4	<0.0001
Former smoker (%)	30.8	1.2	<0.0001
Current smoker (%)	49.7	3.5	<0.0001
Regular exercise (%)^c^	13.5	14.3	0.3367
BMI (kg/m^2^)	24.2 ± 2.9	24.7 ± 3.2	<0.0001
Waist circumference (cm)	83.2 ± 7.6	80.8 ± 9.5	<0.0001
Hypertension history (%)	11.5	14.5	0.0011
Blood pressure (mmHg)			
Systolic blood pressure	116.2 ± 16.2	115.9 ± 19.2	0.4682
Diastolic blood pressure	75.9 ± 11.2	73.2 ± 11.8	<0.0001
Triglyceride (mg/dL)	171.8 ± 112.4	141.4 ± 78.2	<0.0001
Total cholesterol (mg/dL)	192.0 ± 35.1	189.5 ± 34.9	0.0028
HDL-Cholesterol (mg/dL)	43.8 ± 10.0	46.0 ± 10.1	<0.0001
Fasting blood glucose (mg/dL)	85.2 ± 9.4	81.5 ± 8.0	<0.0001
2-h glucose (mg/dL)	113.0 ± 32.5	119.3 ± 29.2	<0.0001
Energy intake (kcal/day)	1992.3 ± 536.0	1833.9 ± 561.8	<0.0001
Energy-adjutsted iron itake (mg/day)	10.3 ± 2.5	9.9 ± 2.7	<0.0001

^a^ T test for continuous variables and Chi-square test for categorical variables

^b^ Mean ± standard deviation

^c^ Regular exercise was defined as ≥3 times per week and ≥30 min per one time.

**Fig 2:**
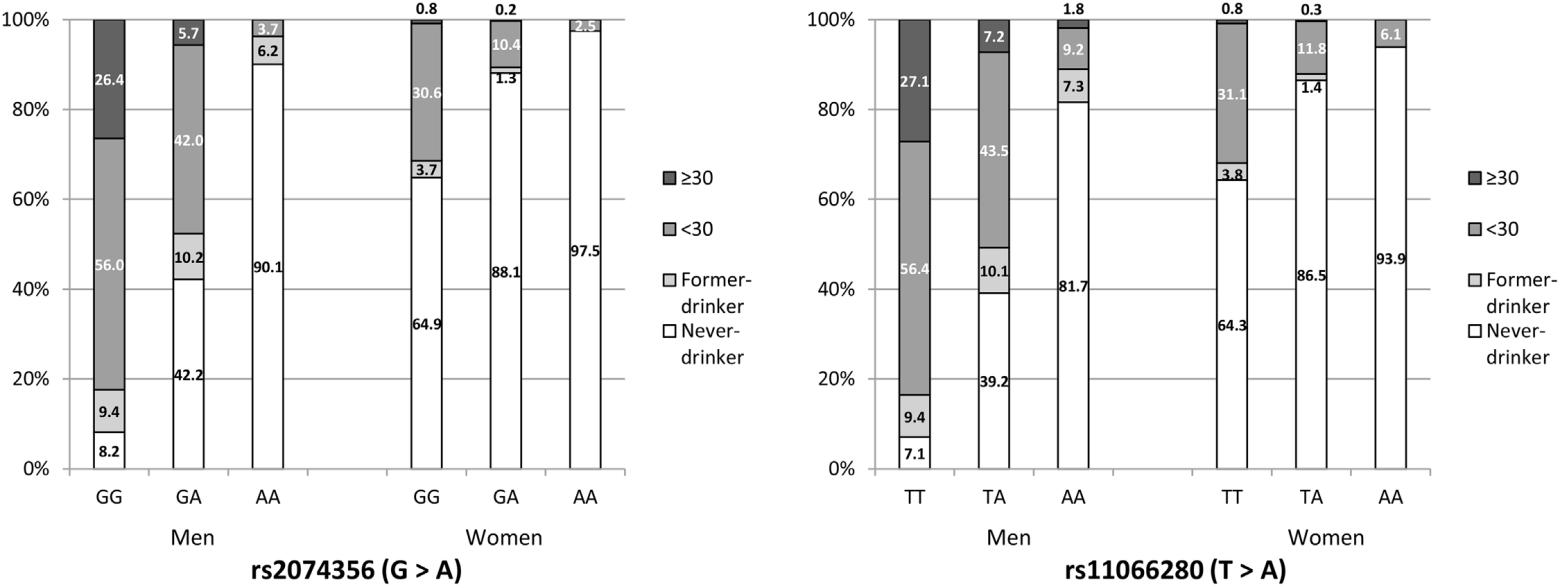
Prevalence of alcohol consumption according to SNP at baseline.

Alcohol consumption substantially differed by C12orf51 genotypes (Fig 2). For both rs2074356 and rs11066280, current-drinker was less frequent with increasing numbers of minor allele. Men and women with AA of rs2074356 and women with AA of rs11066280 did not drink alcohol ≥ 30g/day. There were no female-former drinkers with AA of rs2074356 or AA of rs11066280.

Some characteristics of the study participants across the alcohol consumption groups were selected to detect potential confounders after adjusting for age in Table 2. Among men, age and the proportion of former-smokers decreased across alcohol consumption groups, but the proportion of current-smoker, WC, energy, and iron intake increased. Among women, age and education level decreased, but the proportion of former- and current-smokers increased across the alcohol consumption groups. All variables showing significant differences in means/proportions or significant linear trends were used as covariates in Table 3.

**Table 2:** Age-adjusted characteristics of the study participants by baseline alcohol intake group at baseline ^a^.

			Alcohol consumption, g/day			
Characteristics	Never-drinker	Former-drinker	<30	≥30	*p* for difference	*p* for linear trend
**Men**						
n	613	309	1,658	664		
Alcohol consumption (g/day) ^b^	0 (0, 0)	0 (0, 0)	9.0 (0.2, 29.8)	52.1 (30.1, 303.9)		
Age	51.9±0.3^1^ ^c^	53.3±0.5^1^	50.4±0.2^2^	49.5±0.3^2^	<0.0001	<0.0001
Residential area-Ansan (%)	62.6^1^	54.5^2^	62.4^1^	60.3^12^	0.0279	0.7407
High school or beyond (%)	58.7^12^	57.4^12^	62.8^1^	57.3^3^	0.0172	0.1392
Smoking status (%)						
Former-smoker	27.1^1^	44.7^2^	30.7^1^	27.9^1^	<0.0001	0.0455
Current-smoker	38.0^1^	40.8^1^	51.1^2^	60.9^3^	<0.0001	<0.0001
Regular exercise (%)	11.5	11.9	14.6	13.3	0.2228	0.8006
Family history of diabetes (%)	10.1	9.8	9.3	9.5	0.9340	0.8840
BMI (kg/m^2^)	24.0 ± 0.1	24.3 ± 0.2	24.2 ± 0.1	24.2 ± 0.1	0.2285	0.8465
Waist circumference (cm)	82.3 ± 0.3^1^	83.5 ± 0.4^12^	83.3 ± 0.2^2^	83.7 ± 0.3^2^	0.0051	0.0178
Dietary intake						
Energy (kcal/day)	1,978.2 ± 21.6^12^	2,019.6 ± 30.5^12^	1,969.6 ± 13.1^1^	2,049.4 ± 20.7^3^	0.0080	0.0047
Iron (mg/day)	10.2 ± 0.1^1^	9.9 ± 0.1^1^	10.3 ± 0.6^12^	10.6 ± 0.1^2^	0.0004	0.0002
**Women**						
n	2,598	109	899	23		
Alcohol consumption (g/day)	0 (0, 0)	0 (0, 0)	2.0 (0.1, 29.8)	41.7 (30.4, 93.8)		
Age	52.9±0.2^1^	52.3±0.8^12^	48.7±0.3^3^	47.3±1.8^23^	<0.0001	<0.0001
Residential area- Ansan (%)	52.0	43.2	52.6	65.0	0.1224	0.1515
High school or more (%)	34.8^1^	22.6^2^	33.2^12^	14.8^12^	0.0029	0.0221
Smoking status (%)						
Former-smoker	0.7^1^	5.7^2^	2.0^3^	4.6^123^	<0.0001	0.0385
Current-smoker	1.9^1^	4.7^12^	7.8^2^	13.5^2^	<0.0001	<0.0001
Regular exercise (%)	13.9	14.0	15.2	19.8	0.7033	0.3328
Family history of diabetes (%)	11.2	11.3	11.6	6.4	0.8863	0.5158
BMI (kg/m^2^)	24.7 ± 0.1	25.1 ± 0.3	24.8 ± 0.1	25.2 ± 0.7	0.4121	0.3658
Waist circumference (cm)	80.7 ± 0.2	82.7 ± 0.9	80.9 ± 0.3	82.4 ± 1.9	0.0814	0.3405
Dietary intake						
Energy (kcal/day)	1,826.1 ± 11.0	1,827.9 ± 53.4	1,857.3 ± 18.9	1,821.3 ± 116.3	0.5670	0.8075
Iron (mg/day)	9.9 ± 0.5	9.9 ± 0.3	9.9 ± 0.1	10.1 ± 0.6	0.9745	0.6842

^a^ Values were derived using a general linear model analysis adjusted for age. BMI, Body mass index.

^b^ Median (minimum, maximum).

^c^ Mean ± standard error of the mean (all such values); Mean values within the same row with unlike superscript numbers (1, 2, or 3) were significantly different (P<0.05) according to Tukey’s multiple comparison test.

**Table 3:** Hazard ratios (HRs) and interaction between baseline alcohol consumption and the studied gene polymorphism in relation to type 2 diabetes risk ^a^.

	Alcohol consumption									
	Never-drinker		Former-drinker		<30 g/day		≥30 g/day		*p* for linear trend	*p* for interaction
Men, n	613		309		1,658		664			
Person-years	3,707		1,823		10,101		3,893			
Alcohol consumption (g/day)	0 (0, 0) ^b^		0 (0, 0)		8.97 (0.16, 29.81)		52.09 (30.09, 303.88)			
C12orf51										
rs2074356										
GG	35/191 ^c^	1.00 (ref)	41/220	0.95 (0.60, 1.51)	226/1307	0.82 (0.56, 1.18)	108/617	0.88 (0.59, 1.31)	0.8644	0.2942
GA	48/349	0.69 (0.44, 1.08)	14/84	0.71 (0.38, 1.34)	47/347	0.63 (0.40, 0.98)	5/47	0.52 (0.20, 1.35)	0.4874	
AA	5/73	0.35 (0.14, 0.90)	1/5	0.91 (0.13, 6.70)	0/3	-	0/0	-	0.9988	
*P* for linear trend		0.0035		0.4628		0.0565		0.3559		
rs11066280										
TT	31/155	1.00 (ref)	40/206	0.89 (0.55, 1.45)	209/1237	0.71 (0.48, 1.05)	104/594	0.78 (0.51, 1.19)	0.9648	0.3231
TA	50/369	0.61 (0.38, 0.96)	14/95	0.55 (0.29, 1.05)	64/410	0.65 (0.42, 1.01)	9/68	0.60 (0.28, 1.27)	0.9373	
AA	7/89	0.36 (0.16, 0.82)	2/8	1.23 (0.29, 5.19)	0/10	-	0/2	-	0.9960	
*P* for linear trend		0.0014		0.3570		0.3354		0.4561		
Women, n	2,598		109		899		23			
Person-years	15,772		640		5,562		114			
Alcohol consumption (g/day)	0 (0, 0)		0 (0, 0)		2.03 (0.13, 29.81)		41.68 (30.36, 93.77)			
C12orf51										
rs2074356										
GG	208/1,695	1.00 (ref)	18/79	1.59 (0.98, 2.58)	90/800	0.94 (0.73, 1.22)	2/21	1.21 (0.3, 4.9)	0.9421	0.2350
GA	103/824	1.01 (0.80, 1.28)	0/12	-	11/97	1.25 (0.68, 2.31)	1/2	3.69 (0.51, 26.58)	0.1258	
AA	4/79	0.34 (0.13, 0.93)	0/0	-	0/0	-	0/2	-	0.9984	
*P* for linear trend		0.2348		0.9920		0.4558		0.1992		
rs11066280										
TT	193/1,584	1.00 (ref)	16/94	1.49 (0.89, 2.48)	85/767	0.93 (0.72, 1.22)	2/20	1.36 (0.34, 5.49)	0.8299	0.4868
TA	117/922	1.05 (0.83, 1.33)	2/15	1.20 (0.30, 4.82)	16/126	1.39 (0.83, 2.32)	1/3	2.38 (0.33, 17.07)	0.2612	
AA	5/92	0.39 (0.16, 0.95)	0/0	-	0/6	-	0/0	-	0.9964	
*P* for linear trend		0.3790		0.8370		0.3155		0.3994		

^a^ Values are presented as HRs (95% CIs). HRs were calculated using a Cox proportional hazard model after adjusting for age, residential area, education, smoking status (former-smoker and current-smoker), WC, energy intakes, and iron intakes in men and adjusted for age, education, and smoking status (former-smoker and current-smoker) in women.

^b^ Median (minimum, maximum).

^c^ No. of incident cases/ No. of participants in the cell

For both SNPs, we did not observe interaction effects between alcohol consumption and SNPs (Table 3). Alcohol consumption was not associated with the risk of T2D in the same genotype in both men and women. Participants who were homozygous for the minor allele rarely drank and therefore the effect on T2D incidence could not be detected, but heterozygotes were inversely associated with risk of T2D and interestingly, minor allele homozygote strongly influenced T2D risk reduction among never-drinkers (rs2074356 for AA: HR = 0.35, 95% CI = 0.14–0.90, and *p*-trend = 0.0035 in men and HR = 0.34, 95% CI = 0.13–0.93, and *p*-trend = 0.2348 in women; rs11066280 for AA: HR = 0.36, 95% CI = 0.16–0.82, and *p*-trend = 0.0014 in men and HR = 0.39, 95% CI = 0.16–0.95, and *p*-trend = 0.3790 in women). Homozygous the minor alleles (rs2074356 and rs11066280) decreased the risk of T2D in both men and women, but there was sex-difference in the linear trend. In sensitivity analyses, the linear trend of risk reduction remained (see S1, S2 and S3 Tables). When we used average alcohol consumption to examine the interaction effects on the risk of T2D between alcohol consumption and SNPs, a minor allele of rs11066280 was associated with lower incidence of T2D in men and minor alleles of rs2074356 and rs11066280 were associated with lower incidence of T2D in women (S1 Table). The results remained robust after censoring incident cases of coronary artery disease (CAD), stroke, or cancer during follow-up (S2 Table) and in the analysis using the secondary data set (S3 Table).

## Discussion

In the present study, minor allele homozygote of C12orf51 (rs2074356 and rs11066280) was associated with reduced T2D risk and considerably negatively influenced alcohol consumption. The association of 2 SNPs in C12orf51 with diabetes may not be mediated by alcohol use. In addition, we did not find interaction effects between alcohol consumption and SNPs on the risk of T2D; however, minor allele homozygote of C12orf51 significantly reduced the risk of T2D in never-drinkers.

To the best of our knowledge, the present study provided the first longitudinal evidence on the inverse association between C12orf51 (rs2074356 and rs11066280) and risk of T2D. C12orf51 (rs2074356) was previously reported as a new locus cross-sectionally associated with T2D [14] and glycemic traits [15] using the same dataset of the present study. LDs (D′>0.98) in genotyped or imputed SNPs in C12orf51 and ALDH2 were high in the present study. SNPs in ALDH2 might lead to variations in the production of acetaldehyde dehydrogenase between individuals [26], which could influence the risk of T2D through decreasing heavy alcohol consumption [27, 28]. Therefore, it was possible that signals of C12orf51 stemmed from the region of the ALDH2 gene. However, interestingly, we found that there was no alcohol effect on T2D, but a significant T2D risk reduction with minor allele homozygote, even among never-drinkers. In terms of alcohol consumption behavior, rs671 in ALDH2 was a critical region affecting alcohol drinking behavior [26], although we could not directly compare rs2074356 and rs11066280 in or near C12orf51 the rs671 in ALDH2 in the present study, because rs671 was neither genotyped nor imputed. However, while rs671 did not completely preclude heavy drinking or alcohol dependence due to social pressures in previous studies [29–31], our findings showed that there were no minor allele AA homozygotes at rs2074356 among heavy drinkers (≥ 30g/day) in either men or women. Therefore, it is likely that C12orf51 has a stronger influence on alcohol drinking behavior comparing to ALDH2. Our findings of no association between alcohol consumption and T2D did not support the possible involvement of gene pathways through regulating alcohol intake in development of T2D [32].

ALDH2 has capability to metabolize a number of other short-chain aliphatic aldehydes, some aromatic aldehydes, and polycyclic aldehydes, as well as acetaldehyde [33]. Activated ALDH2 could function as protective enzyme against those toxic agents through reduction of acetaldehyde accumulation and oxidative stress, and as well as protection against lipid peroxidation [33]. The positive association between oxidative stress caused by oxidized LDL and insulin resistance has been observed [34]. Therefore, in never-drinker excluding the impact of alcohol consumption, wild-type ALDH2 may have protective effect against development of T2D. If signals in or near C12orf51 originated from a region of the ALDH2 gene, wild-type C12orf51 should have reduced the risk of T2D in never-drinkers, but minor allele homozygote of C12orf51 significantly reduced the risk of T2D among never-drinkers in the present study. However, Japanese men with minor allele tended to decrease the risk of T2D, independent of alcohol consumption [35]. Thus, we could not assert the independence of C12orf51and ALDH2 in the present study.

Previous studies have reported the association between SNP rs2074356 (C12orf51) with serum gamma-glutamyl transpeptidase (GGT) [16] and waist hip ratio (WHR) [13]. Elevated GGT level and abdominal obesity were known as risk factors for T2D [36–38]. Therefore, to examine that GGT, WHR, and WC may be factors in a mechanism involved by C12orf51, but unrelated to alcohol, the association of GGT, WHR, and WC with those SNPs among never-drinkers. Cells with elevated GGT are vulnerable to oxidative conditions, because reductive properties of thiols of glutathione can change to pro-oxidant. Thus, increasing oxidative stress may increase the risk of T2D [36, 37]. There was no difference in GGT among never-drinkers (mean±SEM of GGT level is presented after adjustment for age according to rs2074356 for GG, GA, and AA, as follows: 25.9±1.7, 27.8±1.3, and 23.6±2.8 in male never-drinkers [*p* = 0.3262]; 17.2±0.4, 16.5±0.6, and 17.9±2 in female never-drinkers [*p* = 0.6433]), whereas there was an overall significant difference in GGT (56.8±1.7, 32.7±2.9, and 24.3±9.1 in men [*p*<0.0001]; 18.3±0.4, 16.3±0.7, and 18.2±2.3 in women [*p* = 0.0449]). The WHR trait showed strong evidence of an association with rs2074356 mapping to chromosome 12q24.13 in the intron of the C12orf51 transcript (effect size = -0.007 ±0.0001; Trend *p* value = 3.3E-08) [13]. We also found that there was no difference in WHR in never-drinkers (mean±SEM of WHR level is presented after adjusted for age according to rs2074356 for GG, GA, and AA, as follows: 0.88±0.004, 0.88±0.003, and 0.88±0.01 in male never-drinkers [*p* = 0.8944]; 0.87±0.002, 0.87±0.003, and 0.85±0.01 in female never-drinkers [*p* = 0.1254]), whereas there was an overall significant difference in WHR (0.89±0.001, 0.88±0.002, and 0.88±0.01 in men [*p* = 0.0001]; 0.87±0.001, 0.86±0.002, and 0.85±0.01 in women [*p* = 0.0379]). In addition, in female never-drinkers, a linear trend in age-adjusted WC according to rs2074356 (81.31±0.22, 80.79±0.31, and 79.40±1.01 in female never-drinkers [*p*-trend = 0.0385]) was found and thus further functional studies of C12orf51 in relation to WC are needed. Homozygous the minor alleles (rs2074356 and rs11066280) decreased the risk of T2D in both men and women, but there was sex-difference in the linear trend. However, it could not be explained due to the lack of functional studies on C12orf51.

The function of the predicted transcript of C12orf51, alternatively referred to as HECTD4, has not been well established to date. However, it is generally accepted that abdominal obesity is a risk factor in the development of T2D [38]. In obese humans, tumor necrosis factor-α (TNF-α) is overexpressed in adipose tissues. TNF-α inhibits serine phosphorylation of insulin receptor substrate-1 and expression of glucose transport protein 4 (GLUT4) causing insulin resistance in adipose tissues [39, 40]. Therefore, one possible mechanism is that C12orf51 polymorphisms (HECTD4) play a role in preventing abdominal obesity by participating in ubiquitination of a certain protein. In the case of the HECT domain containing E3 ubiquitin protein ligase 3 (HECTD3), it might be involved in expression of syntaxin 8, which could be related to GLUT4 function in visceral adipose tissue [41, 42]. In obese patients with T2D, the expression of syntaxin 8 in visceral adipose tissue appeared to be increased, which may result in a decreased level of GLUT4 causing insulin resistance [42]. However, to confirm this mechanism, further functional molecular studies are necessary.

The strengths of our study were large sample size, prospective design, and the use of alcohol consumption and Genome-wide association (GWA) data from a population-based design. However, there were some limitations to this study. First, a validity study for alcohol questionnaire used in the present study was not performed and therefore, we could not rule out the possibility of misclassification of alcohol use. However, HDL cholestc v erol level was steadily increased with alcohol consumption in our additional analysis (data not shown). Furthermore, it has been reported that alcohol consumption assessed by questionnaire may be relatively highly valid [43]. Second, the method of gene selection limited the interpretation of our findings. Although we examined previously reported SNPs to identify the interaction effects between alcohol consumption and SNPs on the risk of T2D, those analyses were part of an exploratory GWA study and thus there was little information on the function in relation to the pathway of T2D development. In addition, because this study population consisted of Korean aged 40 to 69 years, it was limited to generalize the present results to other populations. The minor allele frequencies (MAF) of rs2074356 and rs11066280 were 0.15 and 0.17, respectively, in a Korean population [12], which was similar to MAFs in the Han Chinese (0.12 and 0.16 for MAF of rs2074356 and rs11066280, respectively) [17], but rs2074356 was monomorphic in Europeans [44].

In conclusion, we have identified that, in addition to regulating alcohol consumption, minor allele homozygote of two SNPs in or near C12orf51 (rs2074356 and rs11066280) may decrease the risk of T2D in longitudinal analysis, but this association is unrelated to alcohol consumption. However, further study on the function of C12orf51 is needed to investigate possible mechanisms of T2D development for prevention and/or treatment.

## Supporting Information

S1 Table Hazard ratios (HRs) and interaction between the average alcohol consumption and the studied gene polymorphism in relation to type 2 diabetes risk. (DOCX)

S2 Table Hazard ratios (HRs) and interaction between baseline alcohol consumption and the studied gene polymorphism in relation to type 2 diabetes risk using data that censored incident cases of CAD, stroke, or cancer during follow-up. (DOCX)

S3 Table Hazard ratios (HRs) and interaction between baseline alcohol consumption and the studied gene polymorphism in relation to type 2 diabetes risk using the secondary dataset excluding the participants who did not follow up until 5th survey in 2010. (DOCX)
