# Supplementary material for: No Interaction with Alcohol Consumption, but Independent Effect of C12orf51 (HECTD4) on Type 2 Diabetes Mellitus in Korean Adults Aged 40-69 Years: The KoGES_Ansan and Ansung Study
Source: PLoS One. 2016 Feb 18;11(2):e0149321. doi: 10.1371/journal.pone.0149321 (PMC4758657; doi:10.1371/journal.pone.0149321)
Supplement: S1 Table — (DOCX) [file pone.0149321.s001.docx]

**S1 Table.** **Hazard ratios (HRs) and interaction between the average alcohol consumption and the studied gene polymorphism in relation to type 2 diabetes risk ^a^.**

|  | Average alcohol consumption, g/day | | | | | | | |  |  |
| --- | --- | --- | --- | --- | --- | --- | --- | --- | --- | --- |
|  | 0 | | 0.03-14.98 | | 15.05-29.96 | | ≥30.00 | | *p* for linear trend | *p* for interaction |
| Men, n | 654 | | 1,242 | | 618 | | 730 | |  |  |
| Person-years | 3,741 | | 7,680 | | 3,823 | | 4,280 | |  |  |
| Alcohol consumption (g/day) | 0 (0, 0) ^b^ | | 5.08 (0.07, 14.98) | | 21.5 (15.05, 29.96) | | 46.1 (30.04, 233.63) | |  |  |
| *C12orf51* |  |  |  |  |  |  |  |  |  |  |
| rs2074356 |  |  |  |  |  |  |  |  |  | 0.0615 |
| GG | 43/265 ^c^ | 1.00 | 135/826 | 0.85 (0.60, 1.20) | 100/563 | 0.90 (0.62, 1.30) | 132/681 | 1.07 (0.75, 1.52) | 0.1020 |  |
| GA | 44/317 | 0.78 (0.51, 1.20) | 59/407 | 0.71 (0.48, 1.07) | 4/55 | 0.34 (0.12, 0.96) | 7/48 | 0.88 (0.39, 1.97) | 0.6843 |  |
| AA | 6/72 | 0.47 (0.20, 1.10) | 0/9 | - | 0/0 | - | 0/0 | - | 0.9977 |  |
| *P* for linear trend |  | 0.0107 |  | 0.1608 |  | 0.0714 |  | 0.7223 |  |  |
| rs11066280 |  |  |  |  |  |  |  |  |  | 0.8426 |
| TT | 39/225 | 1.00 | 122/766 | 0.75 (0.52, 1.08) | 95/545 | 0.79 (0.54, 1.17) | 128/656 | 0.97 (0.67, 1.41) | 0.1283 |  |
| TA | 47/343 | 0.69 (0.45, 1.07) | 70/457 | 0.69 (0.46, 1.02) | 9/69 | 0.57 (0.27, 1.18) | 11/73 | 0.82 (0.41, 1.62) | 0.7900 |  |
| AA | 7/86 | 0.41 (0.18, 0.92) | 2/19 | 0.61 (0.15, 2.52) | 0/3 | - | 0/1 | - | 0.8408 |  |
| *P* for linear trend |  | 0.0014 |  | 0.5684 |  | 0.3127 |  | 0.6524 |  |  |
| Women, n | 2,225 | | 1,331 | | 48 | | 25 | |  |  |
| Person-years | 13,026 | | 8,647 | | 289 | | 127 | |  |  |
| Alcohol consumption (g/day) | 0 (0, 0) | | 1.11 (0.03, 14.95) | | 19.20 (15.12, 29.23) | | 36.83 (30.10, 93.77) | |  |  |
| *C12orf51* |  |  |  |  |  |  |  |  |  |  |
| rs2074356 |  |  |  |  |  |  |  |  |  | 0.5253 |
| GG | 184/1,411 | 1.00 | 125/1,133 | 0.78 (0.61, 0.98) | 6/46 | 1.04 (0.45, 2.37) | 3/23 | 1.33 (0.42, 4.23) | 0.5622 |  |
| GA | 93/737 | 0.93 (0.72, 1.19) | 21/194 | 0.88 (0.55, 1.39) | 1/2 | 4.90 (0.68, 35.3) | 0/2 | - | 0.9035 |  |
| AA | 3/77 | 0.24 (0.08, 0.75) | 1/4 | 1.86 (0.26, 13.3) | 0/0 | - | 0/0 | - | 0.0419 |  |
| *P* for linear trend |  | 0.0425 |  | 0.4454 |  | 0.0783 |  | 0.9984 |  |  |
| rs11066280 |  |  |  |  |  |  |  |  |  | 0.3395 |
| TT | 169/1,310 | 1.00 | 118/1,087 | 0.77 (0.61, 0.98) | 6/46 | 1.06 (0.46, 2.42) | 3/22 | 1.48 (0.46, 4.70) | 0.4403 |  |
| TA | 108/827 | 0.99 (0.78, 1.26) | 27/234 | 0.95 (0.63, 1.44) | 1/2 | 5.00 (0.69, 0.99) | 0/3 | - | 0.9091 |  |
| AA | 3/88 | 0.22 (0.07, 0.69) | 2/10 | 1.63 (0.40, 6.58) | 0/0 | 0/0 |  | - | 0.1143 |  |
| *P* for linear trend |  | 0.0822 |  | 0.1989 |  | 0.0783 |  | 0.9987 |  |  |

^a^ Values are presented as HRs (95% CIs). HRs were calculated using a Cox proportional hazard model after adjusting for age, residential area, education, smoking status (former-smoker and current-smoker), WC, energy intakes, and iron intakes in men and adjusted for age, education, and smoking status (former-smoker and current-smoker) in women.

^b^ Median (minimum, maximum).

^c^ No. of incident cases/No. of participants in the cell.
