# Supplementary material for: No Interaction with Alcohol Consumption, but Independent Effect of C12orf51 (HECTD4) on Type 2 Diabetes Mellitus in Korean Adults Aged 40-69 Years: The KoGES_Ansan and Ansung Study
Source: PLoS One. 2016 Feb 18;11(2):e0149321. doi: 10.1371/journal.pone.0149321 (PMC4758657; doi:10.1371/journal.pone.0149321)
Supplement: S2 Table — (DOCX) [file pone.0149321.s002.docx]

**S2 Table.** **Hazard ratios (HRs) and interaction between baseline alcohol consumption and the studied gene polymorphism in relation to type 2 diabetes risk using data that censored incident cases of CAD, stroke, or cancer during follow-up ^a^.**

|  |  | |  | | Alcohol consumption, g/day | | | |  |  |
| --- | --- | --- | --- | --- | --- | --- | --- | --- | --- | --- |
|  | Never-drinker | | Former-drinker | | <30 | | ≥30 | | *p*for linear trend | *p* for interaction |
| Men, n | 613 | | 309 | | 1,658 | | 664 | |  |  |
| Person-years | 3,707 | | 1,823 | | 10,101 | | 3,893 | |  |  |
| Alcohol consumption (g/day) | 0 (0, 0) ^b^ | | 0 (0, 0) | | 8.97 (0.16, 29.81) | | 52.09 (30.09, 303.88) | |  |  |
| *C12orf51* |  |  |  |  |  |  |  |  |  |  |
| rs2074356 |  |  |  |  |  |  |  |  |  | 0.3198 |
| GG | 35/191^c^ | 1.00 | 38/220 | 0.87 (0.54, 1.39) | 216/1,307 | 0.78 (0.54, 1.12) | 100/617 | 0.80 (0.53, 1.19) | 0.7982 |  |
| GA | 45/349 | 0.63 (0.4, 0.99) | 12/84 | 0.60 (0.31, 1.17) | 45/347 | 0.59 (0.37, 0.93) | 5/47 | 0.51 (0.20, 1.32) | 0.6756 |  |
| AA | 5/73 | 0.36 (0.14, 0.92) | 1/5 | 0.86 (0.12, 6.33) | 0/3 | - | 0/0 | - | 0.9991 |  |
| *P* for linear trend |  | 0.0032 |  | 0.4168 |  | 0.0559 |  | 0.4709 |  |  |
| rs11066280 |  |  |  |  |  |  |  |  |  | 0.3604 |
| TT | 31/155 | 1.00 | 37/206 | 0.82 (0.50, 1.33) | 199/1,237 | 0.67 (0.45, 0.997) | 96/594 | 0.71 (0.46, 1.09) | 0.7288 |  |
| TA | 47/369 | 0.56 (0.35, 0.89) | 12/95 | 0.47 (0.24, 0.93) | 62/410 | 0.62 (0.40, 0.97) | 9/68 | 0.58 (0.27, 1.23) | 0.7417 |  |
| AA | 7/89 | 0.36 (0.16, 0.82) | 2/8 | 1.16 (0.28, 4.90) | 0/10 | - | 0/2 | - | 0.9959 |  |
| *P* for linear trend |  | 0.0014 |  | 0.3767 |  | 0.3877 |  | 0.6014 |  |  |
| Women, n | 2,598 | | 109 | | 899 | | 23 | |  |  |
| Person-years | 15,772 | | 640 | | 5,562 | | 114 | |  |  |
| Alcohol consumption (g/day) | 0 (0, 0) | | 0 (0, 0) | | 2.03 (0.13, 29.81) | | 41.68 (30.36, 93.77) | |  |  |
| *C12orf51* |  |  |  |  |  |  |  |  |  |  |
| rs2074356 |  |  |  |  |  |  |  |  |  | 0.3113 |
| GG | 198/1,695 | 1.00 | 17/97 | 1.53 (0.93, 2.52) | 84/800 | 0.92 (0.71, 1.20) | 2/21 | 1.19 (0.30, 4.81) | 0.9965 |  |
| GA | 89/824 | 0.94 (0.73, 1.21) | 0/12 | - | 9/97 | 1.09 (0.56, 2.14) | 1/2 | 3.5 (0.49, 25.24) | 0.1369 |  |
| AA | 3/79 | 0.27 (0.09, 0.86) | 0/0 | - | 0/2 | - | 0/0 | - | 0.9988 |  |
| *P* for linear trend |  | 0.0838 |  | 0.9924 |  | 0.7642 |  | 0.1992 |  |  |
| rs11066280 |  |  |  |  |  |  |  |  |  | 0.6484 |
| TT | 184/1,584 | 1.00 | 15/94 | 1.42 (0.84, 2.42) | 79/767 | 0.91 (0.69, 1.19) | 2/20 | 1.34 (0.33, 5.40) | 0.8913 |  |
| TA | 102/922 | 0.98 (0.77, 1.25) | 2/15 | 1.21 (0.30, 4.87) | 14/126 | 1.27 (0.73, 2.20) | 1/3 | 2.26 (0.32, 16.26) | 0.2848 |  |
| AA | 4/92 | 0.33 (0.12, 0.89) | 0/0 | - | 0/6 | - | 0/0 | - | 0.9969 |  |
| *P* for linear trend |  | 0.1592 |  | 0.8734 |  | 0.4522 |  | 0.3994 |  |  |

^a^ Values are presented HRs (95% CIs). HRs were calculated using a Cox proportional hazard model after adjusting for age, residential area, education, smoking status (former-smoker and current-smoker), WC, energy intakes, and iron intakes in men and adjusted for age, education, and smoking status (former-smoker and current-smoker) in women.

^b^ Median (minimum, maximum).

^c^ No. of incident cases/No. of participants in the cell.
