# Supplementary material for: No Interaction with Alcohol Consumption, but Independent Effect of C12orf51 (HECTD4) on Type 2 Diabetes Mellitus in Korean Adults Aged 40-69 Years: The KoGES_Ansan and Ansung Study
Source: PLoS One. 2016 Feb 18;11(2):e0149321. doi: 10.1371/journal.pone.0149321 (PMC4758657; doi:10.1371/journal.pone.0149321)
Supplement: S3 Table — (DOCX) [file pone.0149321.s003.docx]

**S3 Table.** **Hazard ratios (HRs) and interaction between baseline alcohol consumption and the studied gene polymorphism in relation to type 2 diabetes risk using the secondary dataset excluding the participants who did not follow up until 5th survey in 2010 ^a^.**

|  |  | |  | | Alcohol consumption, g/day | | | |  |  |
| --- | --- | --- | --- | --- | --- | --- | --- | --- | --- | --- |
|  | Never-drinker | | Former-drinker | | <30 | | ≥30 | | *p* for linear trend | *p* for interaction |
| Men, n | 410 | | 215 | | 1,150 | | 438 | |  |  |
| Person-years | 2,965 | | 1,552 | | 8,284 | | 3,142 | |  |  |
| Alcohol consumption (g/day) | 0 (0, 0)^b^ | | 0 (0, 0) | | 9.37 (0.17, 29.64 | | 52.04 (30.37, 231.53) | |  |  |
| *C12orf51* |  |  |  |  |  |  |  |  |  |  |
| rs2074356 |  |  |  |  |  |  |  |  |  | 0.4312 |
| GG | 32/129^c^ | 1.00 | 32/150 | 0.78 (0.47, 1.28) | 196/910 | 0.79 (0.53, 1.16) | 86/407 | 0.8 (0.52, 1.22) | 0.9310 |  |
| GA | 41/230 | 0.68 (0.42, 1.09) | 11/61 | 0.59 (0.30, 1.20) | 39/236 | 0.60 (0.37,0.98) | 4/31 | 0.5 (0.17, 1.43) | 0.4751 |  |
| AA | 5/51 | 0.40 (0.15, 1.02) | 1/4 | 0.91 (0.12, 6.68) | 0/3 | - | 0/0 | - | 0.9985 |  |
| *P* for linear trend |  | 0.0104 |  | 0.5917 |  | 0.0761 |  | 0.4108 |  |  |
| rs11066280 |  |  |  |  |  |  |  |  |  | 0.5123 |
| TT | 29/101 | 1.00 | 31/138 | 0.68 (0.40,1.14) | 180/859 | 0.63 (0.42,0.95) | 83/393 | 0.66 (0.42,1.03) | 0.8258 |  |
| TA | 43/246 | 0.55 (0.34,0.89) | 11/71 | 0.43 (0.21,0.87) | 55/285 | 0.59 (0.37,0.95) | 7/43 | 0.52 (0.22,1.21) | 0.9534 |  |
| AA | 6/63 | 0.32 (0.13,0.77) | 2/6 | 1.16 (0.27,4.90) | 0/6 | - | 0/2 | - | 0.9955 |  |
| *P* for linear trend |  | 0.0010 |  | 0.5010 |  | 0.4740 |  | 0.4892 |  |  |
| Women, n | 1782 | | 70 | | 618 | | 12 | |  |  |
| Person-years | 13175 | | 524 | | 4608 | | 91 | |  |  |
| Alcohol consumption (g/day) | 0 (0, 0) | | 0 (0, 0) | | 1.85 (0.13, 29.81) | | 43.36 (31.95, 71.77) | |  |  |
| *C12orf51* |  |  |  |  |  |  |  |  |  |  |
| rs2074356 |  |  |  |  |  |  |  |  |  | 0.1653 |
| GG | 176/1,166 | 1.00 | 13/62 | 1.36 (0.77, 2.40) | 74/558 | 0.90 (0.68, 1.19) | 1/10 | 0.67 (0.09, 4.79) | 0.5490 |  |
| GA | 81/555 | 0.96 (0.74, 1.26) | 0/8 | - | 10/59 | 1.50 (0.79, 2.86) | 1/2 | 3.98 (0.55, 28.58) | 0.1063 |  |
| AA | 2/61 | 0.20 (0.05, 0.79) | 0/0 | - | 0/1 | - | 0/0 | - | 0.9982 |  |
| *P* for linear trend |  | 0.1051 |  | 0.9920 |  | 0.1544 |  | 0.6320 |  |  |
| rs11066280 |  |  |  |  |  |  |  |  |  | 0.1848 |
| TT | 162/1,099 | 1.00 | 11/59 | 1.23 (0.67,2.28) | 69/533 | 0.89 (0.66,1.19) | 1/9 | 0.81 (0.11,5.78) | 0.6549 |  |
| TA | 95/617 | 1.05 (0.81,1.35) | 2/11 | 1.36 (0.34,5.50) | 15/80 | 1.72 (1.01,2.94) | 1/3 | 2.16 (0.30,15.44) | 0.2994 |  |
| AA | 2/66 | 0.19 (0.05,0.75) | 0/0 | - | 0/5 | - | 0/0 | - | 0.9976 |  |
| *P* for linear trend |  | 0.2360 |  | 0.9671 |  | 0.0688 |  | 0.6669 |  |  |

^a^ Values are presented as HRs (95% CIs). HRs were calculated using a Cox proportional hazard model after adjusted for age, residential area, education, smoking status (former-smoker and current-smoker), WC, energy intakes, and iron intakes in men and adjusted for age, education, and smoking status (former-smoker and current-smoker) in women.

^b^ Median (minimum, maximum).

^c^ No. of incident cases/No. of participants in the cell.
